# Supplementary material for: Novel Driver Strength Index highlights important cancer genes in TCGA PanCanAtlas patients
Source: PeerJ. 2022 Aug 11;10:e13860. doi: 10.7717/peerj.13860 (PMC9375969; doi:10.7717/peerj.13860)
Supplement: Supplemental Information 2 [file peerj-10-13860-s002.zip › COHORTS/genes plots/detailed/2021_8_16_14_9_distribution_events_detailed_males_12.pdf]

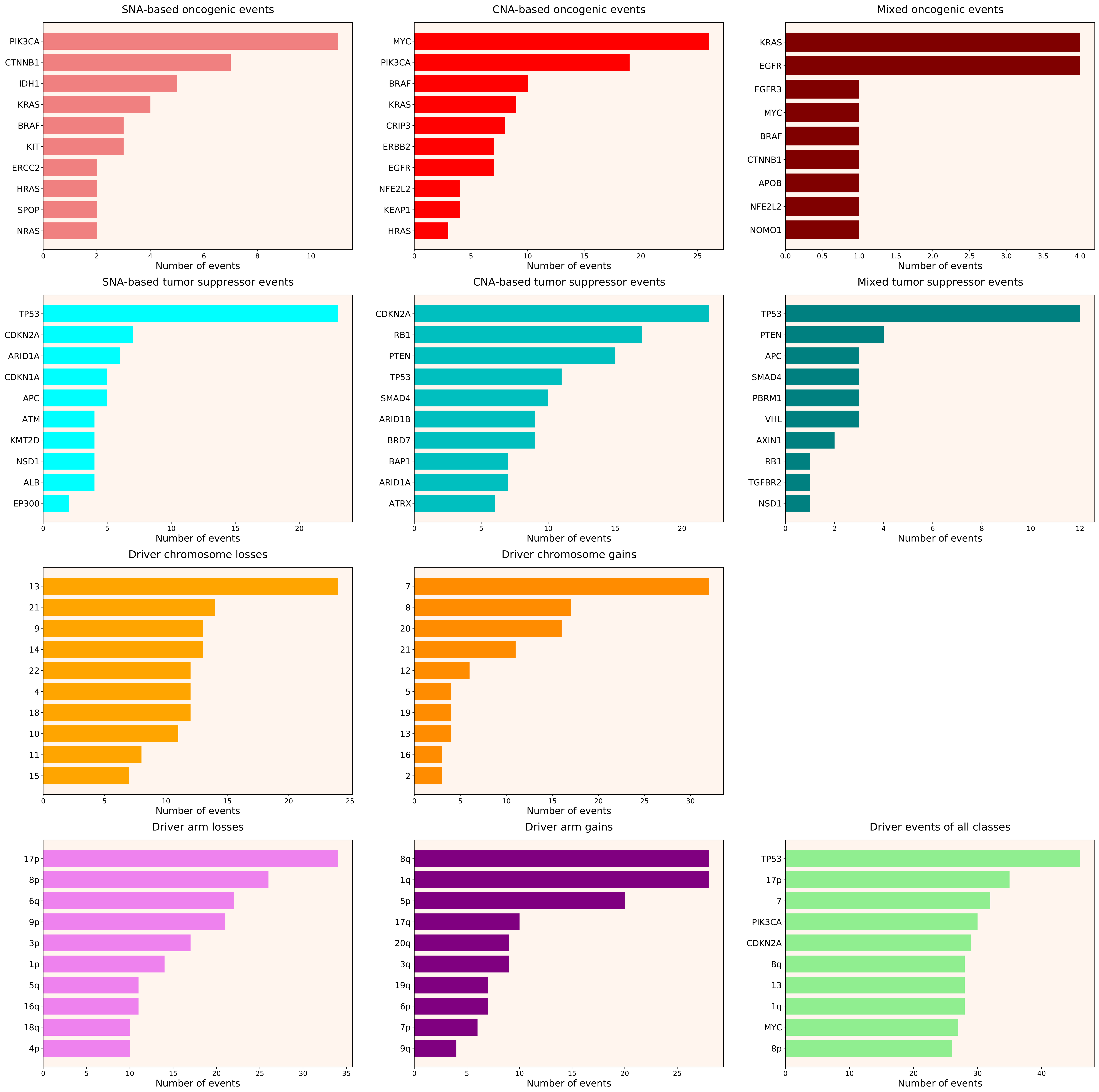

SNA-based oncogenic events
PIK3CA
CTNNB1
IDH1
KRAS
BRAF
KIT
ERCC2
HRAS
SPOP
NRAS
Number of events
CNA-based oncogenic events
MYC
PIK3CA
BRAF
KRAS
CRIP3
ERBB2
EGFR
NFE2L2
KEAP1
HRAS
Number of events
Mixed oncogenic events
KRAS
EGFR
FGFR3
MYC
BRAF
CTNNB1
APOB
NFE2L2
NOMO1
Number of events
SNA-based tumor suppressor events
TP53
CDKN2A
ARID1A
CDKN1A
APC
ATM
KMT2D
NSD1
ALB
EP300
Number of events
CNA-based tumor suppressor events
CDKN2A
RB1
PTEN
TP53
SMAD4
ARID1B
BRD7
BAP1
ARID1A
ATRX
Number of events
Mixed tumor suppressor events
TP53
PTEN
APC
SMAD4
PBRM1
VHL
AXIN1
RB1
TGFBR2
NSD1
Number of events
Driver chromosome losses
13
21
9
14
22
4
18
10
11
15
Number of events
Driver chromosome gains
7
8
20
21
12
5
19
13
16
2
Number of events
Driver arm losses
17p
8p
6q
9p
3p
1p
5q
16q
18q
4p
Number of events
Driver arm gains
8q
1q
5p
17q
20q
3q
19q
6p
7p
9q
Number of events
Driver events of all classes
TP53
17p
7
PIK3CA
CDKN2A
8q
13
1q
MYC
8p
Number of events
